# Differential Temperature Sensors: Review of Applications in the Test and Characterization of Circuits, Usage and Design Methodology

**DOI:** 10.3390/s19214815

**Published:** 2019-11-05

**Authors:** Enrique Barajas, Xavier Aragones, Diego Mateo, Josep Altet

**Affiliations:** Electronic Engineering Department, Universitat Politècnica de Catalunya–BarcelonaTech, 08034 Barcelona, Spain; enrique.barajas@upc.edu (E.B.); xavier.aragones@upc.edu (X.A.); diego.mateo@upc.edu (D.M.)

**Keywords:** complementary metal oxide semiconductor—CMOS—temperature sensor, CMOS analog integrated circuits, differential temperature sensor, built-in sensor

## Abstract

Differential temperature sensors can be placed in integrated circuits to extract a signature of the power dissipated by the adjacent circuit blocks built in the same silicon die. This review paper first discusses the singularity that differential temperature sensors provide with respect to other sensor topologies, with circuit monitoring being their main application. The paper focuses on the monitoring of radio-frequency analog circuits. The strategies to extract the power signature of the monitored circuit are reviewed, and a list of application examples in the domain of test and characterization is provided. As a practical example, we elaborate the design methodology to conceive, step by step, a differential temperature sensor to monitor the aging degradation in a class-A linear power amplifier working in the 2.4 GHz Industrial Scientific Medical—ISM—band. It is discussed how, for this particular application, a sensor with a temperature resolution of 0.02 K and a high dynamic range is required. A circuit solution for this objective is proposed, as well as recommendations for the dimensions and location of the devices that form the temperature sensor. The paper concludes with a description of a simple procedure to monitor time variability.

## 1. Introduction

Nowadays, temperature sensors are integrated with high-density digital integrated circuits, such as microprocessors. The typical applications are power–temperature monitoring to determine if active cooling strategies should be activated, to modulate the microprocessor supply voltage or working frequency, or to ascertain if the workload of a specific microprocessor block can be increased or should be reduced [1,2,3,4]. Moreover, as the temperature is directly proportional to the dissipated power, which is, in turn, dependent on the circuit activity, temperature sensors have been proposed to detect malicious software [5] or hardware Trojans [6].

Can temperature sensors have similar applications in intricate analog designs? Can temperature sensors be used for monitoring purposes of analog circuits built in the same die? The answer to both questions is yes. The temperature increase sensed near an analog circuit depends on its power dissipation, which has two components: one due to the circuit DC bias, another one due to the AC signal. Therefore, the temperature can be seen as a physical magnitude that indirectly monitors the status and activity of an analog circuit. Different types of integrated monitors have been used to regularly check some circuit signatures and activate a feedback procedure to re-adjust bias if circuit behavior is beyond specifications [7,8,9]. Temperature sensors are a specific kind of monitor that are especially attractive for high-frequency analog blocks, since they can get information of the power dissipated by the nearby circuit thanks to the inherent thermal coupling provided by the common silicon substrate, and, therefore, temperature sensors do not touch (i.e., do not electrically load) any node of the circuit under monitoring. Then, there is no need for co-designing the analog circuit and the temperature sensor. For instance, in the context of radio frequency (RF) circuits for wireless communications, temperature sensors have been used to obtain the central frequency and bandwidth in a 60 GHz power amplifier [10] or to perform a structural circuit test in a 2.4 GHz low-noise amplifier [11]. All these monitoring capabilities gain importance in nanometric technologies, where yield is severely compromised due to process–temperature––voltage (PVT) variations [12] and long-time degradation (aging). 

In this review, we first highlight the advantages of differential temperature sensors to monitor dissipated power. Focusing on the test and characterization of analog circuits, Section 2 presents the different strategies to generate a predictable power signature, together with a review of the state of-the-art in thermal monitoring. As a practical application example, the design of a differential temperature sensor to monitor the long-time degradation (aging) of an RF Power Amplifier (PA) is then described. Estimations of the temperature variations to be sensed allow to set demanding targets of resolution (0.02 K) and dynamic range (above 10 K) for this sensor. A circuit solution and measurement approach to provide these demanding characteristics is proposed in Section 3. Based on the analysis of the proposed sensor circuit and estimations on the intrinsic manufacturing variability, a step-by-step methodology to design the components in the circuit is detailed in Section 4. Finally, Section 5 presents the physical implementation of the RF Power Amplifier and sensor circuit in a 0.35 μm CMOS technology, and the procedure to use such a sensor for aging monitoring.

## 2. State-of-the-Art: Thermal Monitoring of Analog Circuits. Principles, Techniques and Sensors

### 2.1. Use of Differential Temperature Sensor

The design of integrated temperature sensors for ambient temperature monitoring has reached an advanced level of maturity [13,14,15,16], with resolutions below 1K and dissipation power below 1 mW. Usually, these sensors are stand-alone in the integrated circuit. Their low power dissipation grants negligible self-heating, fundamental for an accurate ambient temperature measurement. With the advances in device integration, more compact temperature sensors are integrated with complex digital circuits, in order to monitor its working temperature or the presence of hot spots [1,2,3,4,17,18,19,20]. In these cases, the sensor’s output magnitude (usually a digital number) is proportional to the sum of the ambient temperature and the heating generated by the thermal coupling between the temperature sensor and the circuit under monitoring (CUT: the name that we will use for the circuit under monitor when the goal is to perform a test or characterization), i.e.,

(1) $$Output\;Magnitude=S\cdot (T_{Ambient}+R_{TH}\cdot P_{CUT})$$

where *S* is the sensor’s sensitivity, *T_Ambient_* is the ambient temperature, *R_TH_* is the thermal coupling resistance between the CUT and the sensor, and *P_CUT_* is the power dissipated by the CUT. When the goal of temperature monitoring is to perform test/characterization of the CUT, the sensor’s output magnitude should only be proportional to the power dissipated by this circuit, insensitive to ambient temperature changes that might mask the characterization process. Then, in the equation

(2) $$Output\;Magnitude=S_{1}\cdot T_{Ambient}+S_{2}\cdot R_{TH}\cdot P_{CUT}$$

the sensitivity *S*_1_ should ideally be zero, whereas the sensitivity *S*_2_ should be adjusted depending on the expected variation in *P_CUT_* and on the *R_TH_* value. To achieve *S*_1_ to be zero, in [21] a temperature sensor with band pass sensitivity is proposed. As ambient temperature changes are of very low frequency, if temperature changes produced by the *P_CUT_* have a higher frequency, then a frequency-selective sensitivity can provide a sensor output magnitude that is only proportional to *P_CUT_*. Moreover, if the silicon thickness is higher than the silicon thermal characteristic length at the sensitivity central frequency, *R_TH_* only depends on the silicon thermal diffusivity and frequency, and it does not depend on the particular package thermal resistance or IC cooling strategy [22,23].

Another option to achieve null *S*_1_ is the use of differential temperature sensors: sensors with two temperature transducers, named 1 and 2, that sense temperatures *T*_1_ and *T*_2_, respectively, and whose output magnitude is then equal to:

(3) $$Output\;Magnitude=S_{Td}\cdot \left(T_{1}-T_{2}\right)+S_{Tc}\cdot \left(\frac{T_{1}+T_{2}}{2}\right)$$

where *S_Td_* is the sensor differential sensitivity, and *S_Tc_* is the sensor common-mode sensitivity. If *S_Tc_* is zero as a result of design (similar to the common-mode gain in a differential amplifier), ambient temperature changes or any change in the common thermal coupling resistance between the CUT and the temperature transducers (e.g., the activation of a cooling system) do not modify the sensor’s output signal. Then, we can write:

(4) $$Output\;Magnitude≅S_{Td}\cdot (R_{TH1}-R_{TH2})\cdot P_{CUT}$$

where *R_TH_*_1_ and *R_TH_*_2_ are, respectively, the thermal coupling resistances between the CUT and the two temperature transducers. To calculate the difference between both thermal coupling resistances, the thermal analysis can be restricted to the silicon die [24]. The heating of big masses in the package or most of the silicon only affect the common temperature component. Another advantage of this approach is that differential temperature measurements settle faster than absolute temperature measurements, as the thermal steady state has to be reached only in the silicon volume affected by the sensors.

Several circuit topologies can implement differential temperature sensors. A simple circuit topology is a differential amplifier: the classical work [25] shows how a “bad placement” of the transistors that form the input differential pair respect a device with a large power dissipation (“thermal asymmetry”) has a major impact on the amplifier’s output voltage. Following this idea, the first differential temperature sensor was proposed in [26]: its core is a differential amplifier, whose differential pair is, from the electrical bias point of view, identical. Imbalances in the currents flowing within the differential pair are due to a temperature difference between the two transistors that form the differential pair. To achieve this temperature imbalance, one of the devices is physically placed closer to the CUT than the other. Afterwards, current mirrors and, eventually, a high impedance node, transform these current imbalances into output voltage changes. Further details of this circuit are discussed in Section 3.1.

### 2.2. Circuit under Test Biasing Strategies: DC, Homodyne and Heterodyne Temperature Measurements

**Figure 1:**
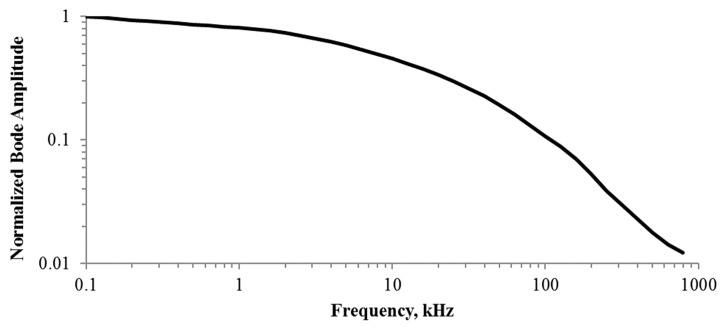
Example of thermal coupling. Normalized frequency response. CUT is a MOS transistor (dimensions: *W* = 100 µm, *L* = 10 µm) at a 25 µm distance from the temperature transducer. Extracted from [27].

Two physical mechanisms link the CUT and the sensor’s temperature transducers: thermal coupling and the Joule Effect. Thermal coupling is the physical phenomena that relates the temperature increase at one point of the silicon surface (i.e., the location of a temperature transducer) with the power dissipated by the CUT. A key property is that, for low-power dissipation magnitudes, it behaves as a linear low pass filter, attenuating the high-frequency components. Figure 1 shows the thermal coupling frequency response measured in [27]. It was obtained using a differential temperature sensor whose temperature transducers were placed 240 µm apart, with the CUT being a MOS—Metal Oxide Semiconductor—transistor placed at 25 µm from temperature transducer 1. There, the attenuation at 100 kHz is approximately one order of magnitude higher than that experienced at 100 Hz, whereas at 1 MHz, the attenuation is approximately two orders of magnitude higher. As the distance between the CUT and the temperature transducer increases, the attenuation becomes stronger, which reduces the filter cut-off frequency [28].

**Figure 2:**
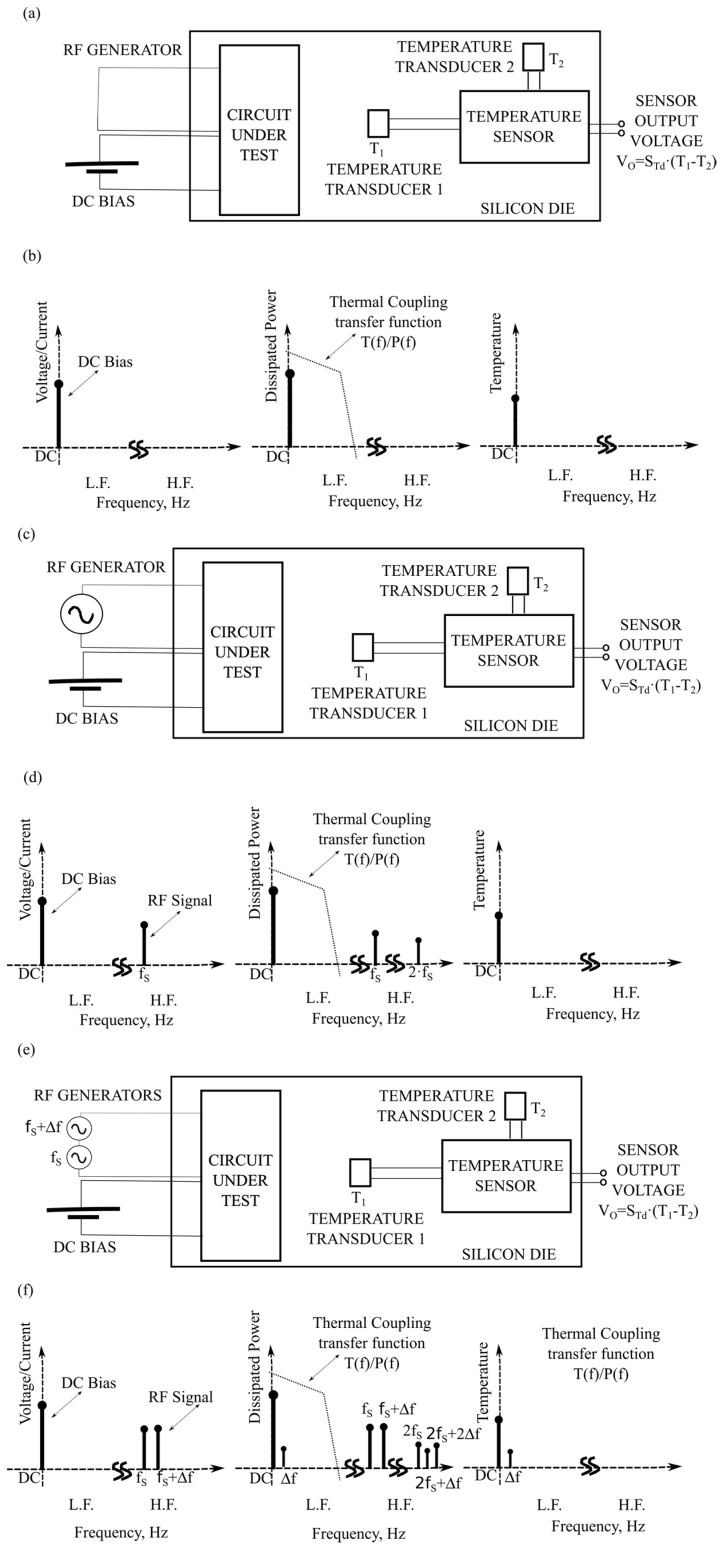
Three different techniques to measure CUT properties by thermal measurements: (**a**,**b**), by applying only DC and measuring DC temperature; (**c**,**d**), by applying DC and one signal tone and measuring DC temperature—Homodyne technique; and (**e**,**f**), by applying DC and two-signal-tones-separated Δ*f* and measuring the temperature spectral component at Δ*f*—Heterodyne technique.

To observe figures of merit of circuits working within the GHz range when temperature measurements are at low frequency, we should properly bias and drive the input of the CUT. Figure 2 summarizes the three strategies that can be used to monitor CUT figures of merit through temperature measurements: DC measurements (Figure 2a,b), homodyne measurements (Figure 2c,d) and heterodyne measurements (Figure 2e,f). The figure shows a silicon die where an analog CUT is sharing the silicon die with a differential temperature sensor. For simplicity, let us assume that the CUT is a linear amplifier. In the figure, the CUT has two input ports: one to perform the DC bias and another to enter the high-frequency signal that is to be amplified. Focusing now on the temperature sensor, its goal is to monitor a property of the CUT, e.g., its gain.

**DC temperature measurements** consist of DC biasing the CUT (Figure 2a,b). The CUT does not have any AC signal applied to its signal input. Therefore, only DC electrical signals are within the CUT, which generates DC power dissipation and DC temperature increases. These measurements have been used to perform the structural test of RF circuits [11] and to assert the RF performances of RF amplifiers [29]: high-frequency figures of merit can be derived from DC measurements since small-signal device parameters (such as MOS transconductance or MOS output resistance) depend on the DC operating point.

**Homodyne temperature measurements** (Figure 2c,d). The CUT is DC-biased, and its input is driven with a single RF sinusoidal tone of frequency *f_S_*. The voltage and current flowing through each CUT device can be written as:

(5) $$\begin{array}{c} v\left(t\right)=V_{DC}+A\cdot \cos\left(2\pi f_{s}t\right)\\ i\left(t\right)=I_{DC}+B\cdot \cos\left(2\pi f_{s}t\right) \end{array}$$

Then, thanks to the Joule Effect, the power dissipated by these devices generates low-frequency and high-frequency signatures given by the following:

(6) $$P\left(t\right)=\left[V_{DC}I_{DC}+\frac{AB}{2}\right]+\left[BV_{DC}+AI_{DC}\right]\cos\left(2\pi f_{s}t\right)+\frac{AB}{2}\cos\left(4\pi f_{s}t\right)$$

If *f_S_* is in the RF or mmW—millimeter wave—range, because of the thermal inertia of the coupling (see frequency response of the thermal coupling in Figure 1), only the average power dissipated will generate a temperature difference *T*_1_ *− T*_2_:

(7) $$T_{1}-T_{2}\approx (R_{TH1}-R_{TH2})\cdot \left[V_{DC}I_{DC}+\frac{AB}{2}\right]=R_{THD}\cdot P_{DC}$$

where *R_THD_* is the differential thermal coupling resistance between the CUT device and the temperature transducers and *P_DC_* is the DC component of the power in Equation (6). Equation (6) shows that the DC power dissipated by the CUT depends on both the DC CUT bias and the high-frequency electrical signals present in the CUT. Interestingly, DC temperature measurements provide information of the high-frequency CUT behavior, regardless of its working frequency *f_S_*. This property allows the generation of a multi-standard monitoring circuit. As an example, the same differential temperature sensor has been used in [30,31] to monitor the power delivered to the load and the efficiency of a 2.4 GHz amplifier and in [10,32] to extract the central frequency and the 3 dB bandwidth of a 60 GHz power amplifier.

**Heterodyne temperature measurements** (Figure 2e,f). The CUT is DC-biased and its input driven with a two-tone RF sinusoidal signal of equal amplitude at *f_S_* and *f_S_ +* Δ*f*, respectively. The voltage and current of each CUT device can be written as follows:

(8) $$\begin{array}{c} v\left(t\right)=V_{DC}+A\cdot \left[\cos\left(2\pi f_{s}t\right)+\cos\left(2\pi \left(f_{s}+\Delta f\right)t\right)\right]\\ i\left(t\right)=I_{DC}+B\cdot \left[\cos\left(2\pi f_{s}t\right)+\cos\left(2\pi \left(f_{s}+\Delta f\right)t\right)\right] \end{array}$$

The power dissipated by the CUT generates several spectral components (see Figure 2f). Among them, the two most interesting are

(9) $$P\left(t\right)=\left[V_{DC}I_{DC}+AB\right]+AB\cos\left(2\pi \Delta ft\right)+\mathrm{Higher}\;\mathrm{frequency}\;\mathrm{terms}$$

The low-frequency components generate a measurable temperature increase

(10) $$\begin{array}{c} T_{1}\left(t\right)-T_{2}{\left(t\right)}_{}≅R_{THD}\cdot \left[V_{DC}I_{DC}+AB\right]+Z_{THD}\left(\Delta f\right)\cdot AB\cdot \cos\left(2\pi \Delta ft\right)\\ =R_{THD}\cdot P_{DC}+Z_{THD}\left(\Delta f\right)\cdot P_{\Delta f}\left(t\right)=T_{DC}+T_{\Delta f}\left(t\right) \end{array}$$

where $P_{\Delta f}\left(t\right)$ is the spectral component at Δ*f* of the power in Equation (9) and *Z_THD_*(Δ*f*) is the differential thermal coupling impedance between the CUT and the temperature transducers at the frequency Δ*f*, which is complex. The DC temperature increase, *T_DC_*, depends on both the DC bias and the power of the RF stimuli. On the other hand, according to Equation (10), there is an AC component of the temperature increase generated at Δ*f*, *T*_Δ*f*_(*t*), whose amplitude depends only on the power of the test RF signals presents in the CUT and whose frequency is independent of *f_S_*. The goal of heterodyne temperature measurements is to derive CUT figures of merit from measurements of this temperature spectral component *T*_Δ*f*_(*t*) [33]. 

### 2.3. Differential Temperature Sensors. State-of-the-Art

Table 1 lists some of the differential temperature sensors reported in the literature. The table compares the following fields: technology, devices that form the differential pair, CUT with its frequency of operation, figure of merit observed and CUT driving technique. The devices that form the differential pair are bipolar transistors if the technology is BiCMOS or, if the technology is MOS, they can be either bipolar parasitic transistors, or directly MOS transistors (although none of the works listed in Table 1 uses this option). Regarding the parasitic bipolar transistors, they can be either lateral, vertical with collector connected to ground, or vertical bipolar parasitic transistors in a nwell/pwell/n+diffusion structure (MOS technology must be triple-well). 

## 3. Differential Temperature Sensor with High Sensitivity and High Dynamic Range

As a practical application example, the following sections describe the design process of a differential temperature sensor to monitor the long-time degradation (aging) of an RF Power Amplifier (PA). Among the different circuits in a wireless transceiver, the PA circuit is particularly affected by aging degradation. Details of the 2.4 GHz PA designed in a 0.35 µm CMOS technology are later given in Section 5. The required sensor sensitivity for this application depends on the power dissipated by the PA circuit, amount of aging degradation expected, distance between the dissipating element and the transducer, and IC physical characteristics, among others. Expected temperature variations can be estimated with electro-thermal simulations of the PA circuit together with a thermal model of the IC. Using this analysis, presented in [37], a resolution of only 0.02 K is targeted in this case. On the other hand, the sensor dynamic range should cover not only the temperature variations produced by aging but also those related to the DC—bias point—dissipation and also account for other sources of variability. Qualitative discussion on these requirements, together with the circuit proposal to achieve both high sensitivity and high dynamic range, is presented in this section, while the quantitative analysis and design are developed in Section 4. 

### 3.1. Basic Differential Temperature Sensor

**Figure 3:**
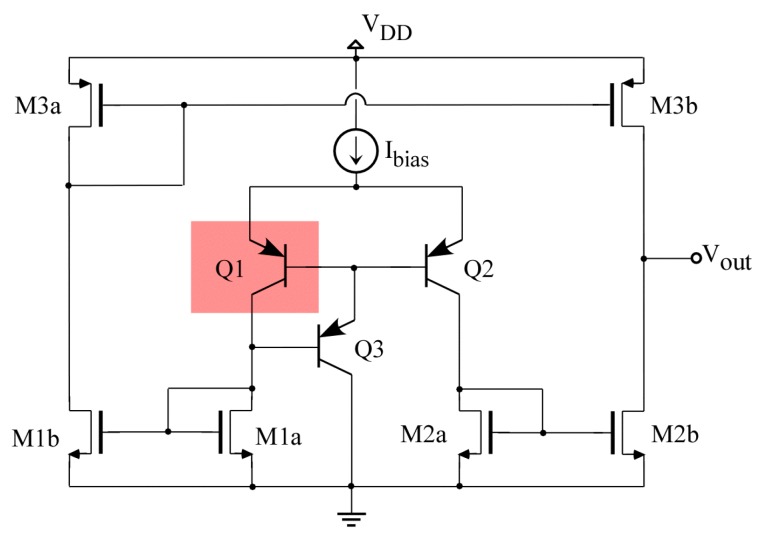
Schematic of the core circuit of the temperature sensor. Transistor Q1 is placed by the CUT and away from Q2 and the rest of the sensor.

Figure 3 shows the schematic of the core sensor [26]. The working principle of the sensor is as follows: Q1 and Q2 are bipolar transistors acting as temperature transducers, producing a current that depends on their temperature difference. If both transducers Q1 and Q2 are at the same temperature, the currents that flow through each branch of the differential pair are identical. Currents are then copied to the output branch through the current mirror structures M1a-M1b, M2a-M2b and M3a-M3bA. If mirrors are perfectly matched, then the current injected by transistor M3b is equal to the current sunk by M2b, and the output voltage is preserved at its quiescent value (nominally *V_OUT,Q_ = V_DD_/2*). Transistor Q1 (gray area) is placed by the CUT and then away from Q2 and the rest of the sensor. A temperature imbalance Δ*T_Q_ = T_Q_*_1_ *− T_Q_*_2_ produced by heating Q1 generates a mismatch in these currents, *I_Q_*_1_ *− I_Q_*_2_ *=* Δ*I_Q_*, and the current mirrors translate that current imbalance into variations of the output voltage. With *K_M_* being the gain produced by the current mirrors and *R_OUT_* the small-signal resistance at the output node, output voltage variations can be related to the temperature difference through the expression [23]:

(11) $$\Delta V_{OUT}=R_{OUT}\Delta I_{OUT}=R_{OUT}K_{M}\Delta I_{Q}=R_{OUT}K_{M}S_{BJT}\Delta T_{Q}$$

where *S_BJT_* denotes the “thermal transconductance” produced by the bipolar transistors, *S_BJT_* = Δ*I_Q_/*Δ*T_Q_*. Thermal transconductance, with units (A/K), is defined equivalently in this thermal sensor to the classical electrical transconductance with units (A/V). Note also that the change of temperature Δ*T* can be expressed in K or °C. In this work, we use ΔT = (K), (A/K) when talking about thermal transconductance and (V/K) when talking about thermal sensitivity (or thermal gain) of the whole sensor. 

**Figure 4:**
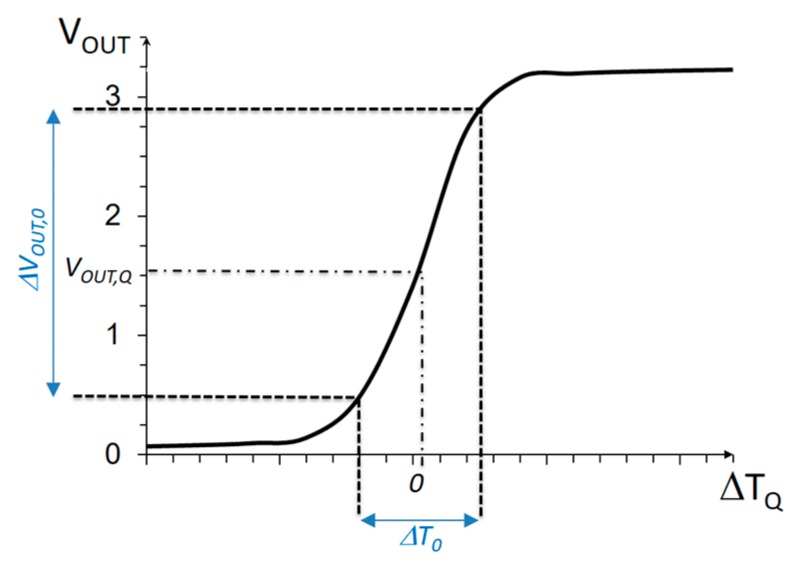
Typical characteristic curve of the core temperature sensor in Figure 3.

Figure 4 shows the typical output curve produced by the former sensor circuit, as a function of the temperature difference in the transducers, Δ*T_Q_*. Note, the sensor shows a roughly linear characteristic for a range of temperatures, Δ*T*_0_. A basic characteristic of the sensor is its sensitivity or gain, which, based on the results in Figure 4, can be defined as Equation (12). The larger the gain, the smaller the temperature variations that can be detected inside the linear region. Correspondingly, another basic characteristic of the circuit is its dynamic range, or the maximum temperature difference that can be measured working in the linear region, which, in Figure 4, equals approximately Δ*T*_0_. Similar to any electrical–electrical amplifier, the larger the gain, the smaller the dynamic range.

(12) $$Sens={\frac{dV_{OUT}}{dT}|}_{V_{OUT,Q}}\approx \frac{\Delta V_{OUT,0}}{\Delta T_{0}}$$

### 3.2. Extending the Dynamic Range

**Figure 5:**
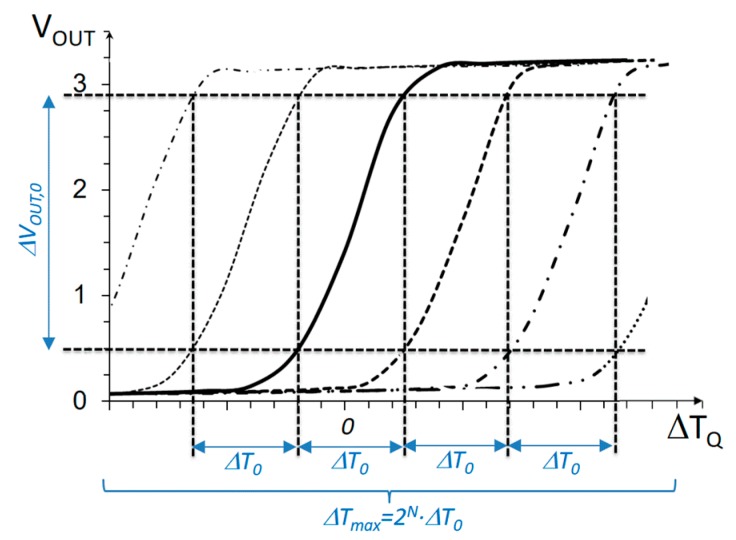
Characteristic curve of a differential temperature sensor with an extended dynamic range.

In order to achieve large dynamic range and high sensitivity at the same time, a modification to the above basic differential sensor is proposed [38]. Figure 5 sketches the purpose of the proposed modification. The target is to introduce shifts in the transfer curve of the circuit with a digital control, such that the total range of detectable temperatures is extended. If, for example, the transfer curves are shifted Δ*T*_0_ with respect to each other, and a total of 2*^N^* possible shifts are introduced (for an *N*-bit binary control), the total dynamic range will be extended to Δ*T_max_ =* 2*^N^·*Δ*T*_0_, while the sensitivity is still that of the core circuit in Equation (12). 

**Figure 6:**
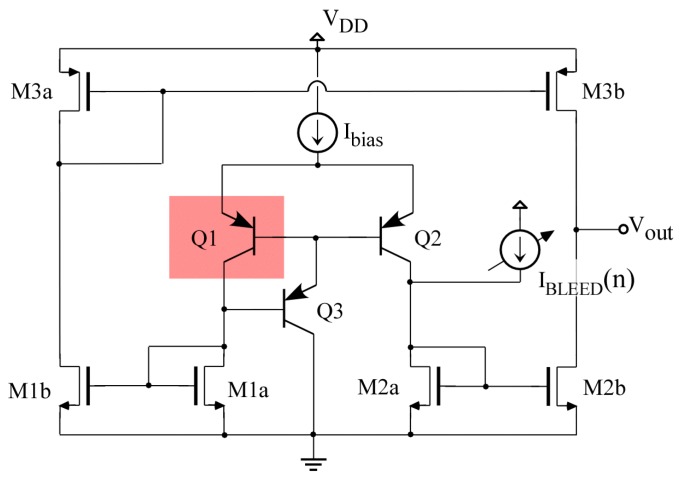
Schematic of the temperature sensor with current bleeding for extended dynamic range and variability compensation.

The proposed dynamic range extension approach can be implemented by adding or subtracting digitally controlled currents to those produced by the transconductors Q1 and Q2. Specifically, if only positive temperature differences are to be detected (transducer Q1 will always be heated as a consequence of power dissipation in the CUT), then a digitally controlled bleeding current *I_BLEED_*(*n*) must be subtracted from the current produced by Q1, as evidenced from Equation (13). Note that, because of the differential sensor topology, subtracting a current from Q1 is equivalent to adding a current to Q2 in the opposite branch, thus the modification sketched in Figure 6 will be implemented in practice.

(13) $$\Delta V_{OUT}=R_{OUT}\Delta I_{OUT}=R_{OUT}K_{M}\left(\Delta I_{Q}-I_{BLEED}\left[n\right]\right)=R_{OUT}K_{M}\left(S_{BJT}\Delta T_{Q}-I_{BLEED}\left[n\right]\right)$$

### 3.3. Improving Resolution

The measurement procedure for the sensor solution described above would then consist of setting the digital code *n* in such a way that the output voltage is moved into the linear region of the transfer curve (coarse tuning), and then, the voltage deviation from the quiescent value Δ*V_OUT_* = *V_OUT_* − *V_OUT,Q_* (fine measure) is measured. From Equation (13), the actual temperature gradient Δ*T_Q_* in the circuit can be obtained from *n* and Δ*V_OUT_* as follows:

(14) $$\Delta T_{Q}=\frac{1}{S_{BJT}}\left(\frac{\Delta V_{OUT}}{R_{OUT}K_{M}}+I_{BLEED}\left[n\right]\right)=\frac{\Delta V_{OUT}}{Sens}+\frac{I_{BLEED}\left[n\right]}{S_{BJT}}$$

**Figure 7:**
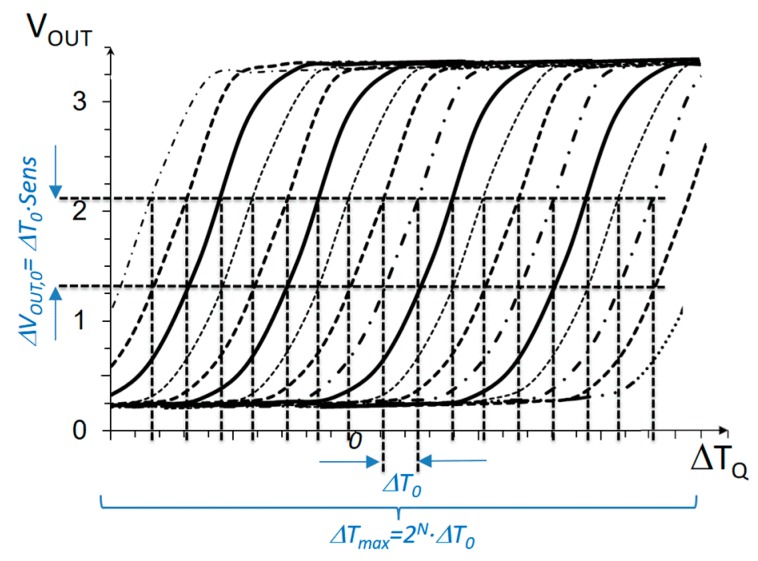
Characteristic curve of a differential temperature sensor with extended dynamic range and improved resolution (smaller Δ*T*_0_*)*.

One limitation of the above measurement procedure is that the fine measure depends on the sensor sensitivity *Sens*, which is not constant within the Δ*T*_0_ range. In other words, as can be observed in Figure 4, the non-linearity of the transfer curve would limit the actual resolution of the measurement. This is a limitation in this application, since temperature variations below 0.1 K are to be detected. From observation of the transfer curve in Figure 4, the effects of non-linearity in the Δ*V_OUT_*(Δ*T_Q_*) relation can be reduced if a smaller linear output voltage range Δ*V_OUT_*_,0_ is set (restricting to a smaller section of the transfer curve). Evidently, this will decrease the Δ*T*_0_ linear range, as shown in Figure 7; then, to preserve the same total dynamic range Δ*T_max_*, a larger number of bits controlling *I_BLEED_*(*n*) will be necessary. 

Taking this reduction of the linear range to the limit, Δ*T*_0_ could be reduced down to the targeted resolution of the measurement. If the digitally controlled bleeding current source is able to bring the output within the targeted resolution, the fine measurement provided by the output voltage is then unnecessary, and the first term in Equation (14) can be ignored. Then, the temperature measurement will be obtained directly from the digital code *n*, with a resolution limited by the linearity and noise of the *I_BLEED_*(*n*) current. The measurement procedure will consist of setting the digital code *n* that takes the output voltage within the resolution of the sensor, i.e.,

(15) $$V_{OUT}\in \left\{V_{OUT,Q}-\frac{1}{2}\Delta T_{0}\cdot Sens\;,\;V_{OUT,Q}+\frac{1}{2}\Delta T_{0}\cdot Sens\right\}$$

and then, the temperature gradient Δ*T_Q_* in the circuit is given as

(16) ΔTQ=IBLEED[n]SBJT

### 3.4. Correction of Variability Offsets

**Figure 8:**
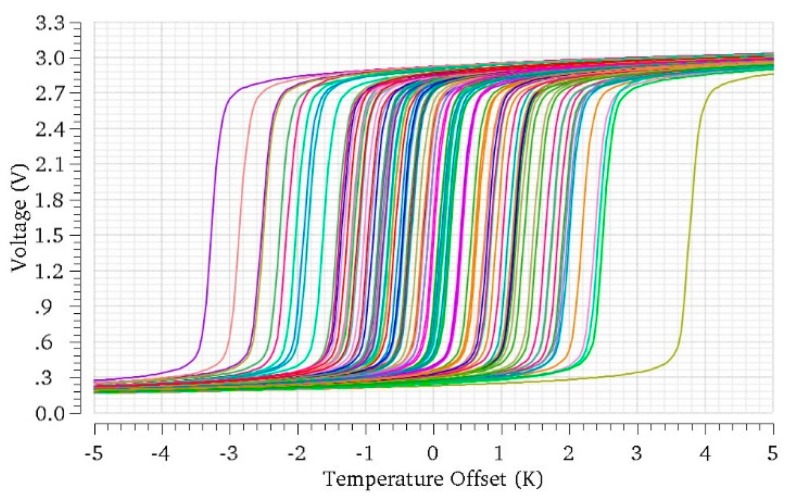
Simulation of the core sensor in Figure 6, at balance (Δ*T_Q_* = 0 K) for several occurrences of the random variations of the components in the circuit.

The above explanation of the sensor operation starts from the assumption that, when both transducers are at the same temperature (Δ*T_Q_* = 0 K), the core circuit is balanced and the output voltage is the targeted *V_OUT_*_,*Q*_. Unfortunately, in practical implementations, this will rarely happen. On one side, variability associated to the manufacturing process will produce mismatches between transconductors Q1 and Q2, or between the transistors in the different current mirrors. These mismatches will produce current imbalance in the output branches and thus offsets in the transfer curve, which can drive the sensor out of its linear region even if Δ*T_Q_* = 0 K. IC manufacturers provide statistical models of component variability, and this way, they can be accounted for during the circuit design. As an example, Figure 8 shows several characteristic curves obtained after simulation of the core sensor in Figure 6, with Δ*T_Q_* = 0 K, for 100 occurrences of the random variations of the components in the circuit, for an ambient temperature of 300 K. Note, how the range of variability (referred to the input) is much larger than the linear range of the sensor Δ*T*_0_; thus, the sensor output can easily be saturated at balance.

Component variability produced by manufacturing is not the only cause of offsets. Other neighboring circuits may produce temperature gradients in the IC, thus provoking a temperature imbalance in the circuit even if the amplifier to be sensed is off. Both manufacturing and external temperature gradients can be corrected with the help of bleeding currents, as shown in Figure 6. The only difference is that now both positive and negative offsets must be corrected, thus both added and subtracted bleeding currents are necessary.

## 4. Design Methodology for the Temperature Sensor

### 4.1. Sensor Core

**Figure 9:**
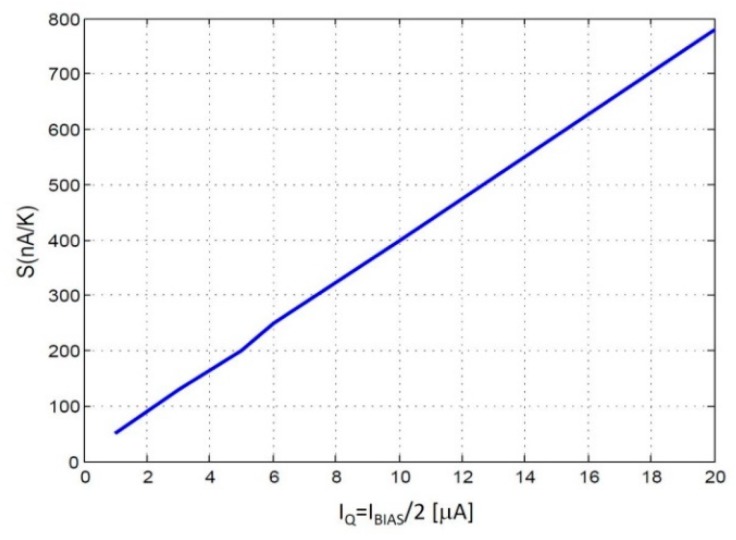
Thermal transconductance of the lateral PNP transistors, *S_BJT_*, in function of the DC current set by *I_BIAS_*.

Design of the temperature sensor starts with the core circuit in Figure 3. The main target for this core sensor is to maximize its sensitivity, while achieving reasonable dynamic performance (slew-rate and bandwidth). As shown in Equation (11), sensitivity depends on the thermal transconductance produced by the bipolar transistors, *S_BJT_ =* Δ*I_Q_/*Δ*T_Q_,* the gain *K_M_* of the current mirror structures, and the small-signal output resistance *R_OUT_.* The 0.35 μm technology selected only allows lateral PNP bipolar transistors with the dimensions already set, thus the only tuning value for the bipolar transistors is the DC-bias current, set by current source *I_BIAS_* in Figure 3. Figure 9 shows how BJT thermal transconductance increases linearly with the DC current, and can be quantified as *S_BJT_* ≈ *I_Q_*·40 nA/K, with current *I_Q_* expressed in μA.

Observing the dependence in Figure 9, it appears that the larger the bias current, the better the sensitivity. But a larger DC current will reduce the output resistance *R_OUT_*, which shows a roughly inverse relationship with the current. Increasing the gain of the current mirrors produces the same effect, since the current would be multiplied by a factor *K_M_*, producing a similar decrease in the output resistance *R_OUT_.* Therefore, in a first-order analysis, the sensor sensitivity in Equation (11) would to be independent of *I_BIAS_*, due to the complementary dependences of *S_BJT_* and *R_OUT_*. In practice, some dependence of the sensor sensitivity with *I_BIAS_* is expected, set by second-order relationships. Note that, according to the measurement procedure proposed in Section 3.3, the total sensor sensitivity *Sens* will be of smaller importance, with the BJT thermal transconductance being a more critical parameter.

Regarding the dynamic characteristics of the sensor, they will be set by the output resistance *R_OUT_* and the DC current at the output branch. The larger the DC current, the larger the slew-rate. Moreover, the larger the DC current, the smaller the output resistance *R_OUT_*, and the larger the bandwidth, however, the smaller the sensitivity. Since the purpose of the sensor is to detect slow variations produced by long-time degradations of the power amplifier, high measurement speed is not required, thus relatively low slew-rate values would be enough for this application. Regarding bandwidth, it must be large enough to accommodate the frequency difference between input tones in heterodyne measurements; thus, a minimum value of 10 kHz (typical thermal bandwidth for usual distances between CUT and Q1, see Figure 1) is targeted. 

**Figure 10:**
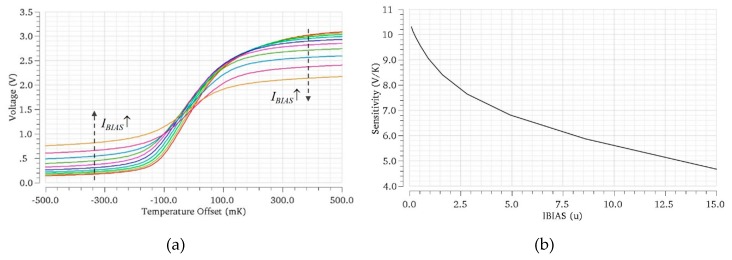
(**a**) Transfer curves of the core sensor for different *I_BIAS_* currents and (**b**) sensitivity dependence on *I_BIAS_*.

Taking into account all the above considerations, the core sensor circuit has been designed. Given the complementary effect of the gain of the current mirrors, a ratio 1:1 has been selected for simplicity. In order to maximize the output resistance *R_OUT_* and thus, the sensitivity, transistors have been sized with channel lengths well above the minimum allowed by the technology. Regarding the dependence with DC current, Figure 10a shows transfer curves for different *I_BIAS_* values, and the sensitivity (slope at *V_OUT,Q_ = V_DD_/2*, Equation (12)) measured in these curves (Figure 10b), at an ambient temperature 300 K. It can be observed how sensor sensitivity increases with smaller *I_BIAS_* values. Sensitivity dependence on the common-mode ambient temperature is $0.01\cdot \left(T_{amb}-300K\right)\left[V/K\right]$. Finally, Table 2 summarizes the performance of the basic sensor, in two different bias scenarios, *I_BIAS_* = 1 µA and *I_BIAS_* = 6 µA. Slew-rate and BW are evaluated with the sensor loaded with an OpAmp-based voltage follower, used to drive the voltages to the IC output. The slew-rate values—tens of kV/s—are considered to be sufficient according to the above discussion, while sensor BW exceeds the targeted value, even for the smallest *I_BIAS_* current considered. Note that an optimized design could further reduce the loading of the sensor output and thus improve the dynamic characteristics, if needed.

### 4.2. Current Bleeding

As explained in Section 3, a digitally controlled bleeding current is necessary to obtain a large dynamic range, as well as to correct offsets produced by different sources of variability. Moreover, a linear relationship of the current vs. N-bit controlling code is required, since the temperature reading (Equation (16)) relies on that. Therefore, the circuit implementing the bleeding current source is just a current-steering N-bit digital-to-analog (DAC) converter. The basic characteristics of this current-steering DAC need now to be determined: the number of bits *N*, and its resolution or current produced by the Least-Significant Bit (LSB), *I_LSB_*. Full-Scale current *I_FS_*, is then given by expression

(17) $$I_{FS}=2^{N}\cdot I_{LSB}$$

From Equation (16), the *I_LSB_* or minimum current must produce an offset in the transfer curve not larger than the targeted measurement resolution, ΔT0≥ILSBSBJT. As explained in Section 3, a resolution of at least Δ*T*_0_ = 0.02 K has been decided for the temperature sensor. The targeted *I_LSB_* will thus depend on the *I_BIAS_* current selected but can be approximated as follows:

(18) ILSB≤ΔT0·SBJT≈ΔT0·IQ[μA]·40nAK=0.4·IBIAS[μA] nA

Once the DAC resolution is set, the number of bits *N* will be determined from the maximum offset to be produced by the bleeding current. As explained in Section 3.2, this offset must be sufficient to cover the targeted dynamic range or maximum temperature difference to be measured Δ*T_max_*. From the analysis in [37], temperature variations below 1 K are expected as a consequence of the aging degradation of this PA under test. However, the dynamic range must also be enough to cover the temperature increase produced by the DC bias of the PA and the RF signal component. Those temperature differences will be much larger than those produced by device degradation over time. Again, electro-thermal simulations of the PA circuit together with thermal models of the IC and package have been used to estimate that a maximum temperature variation of 10 K is expected as a consequence of the operation of the PA, in the worst situation.

**Figure 11:**
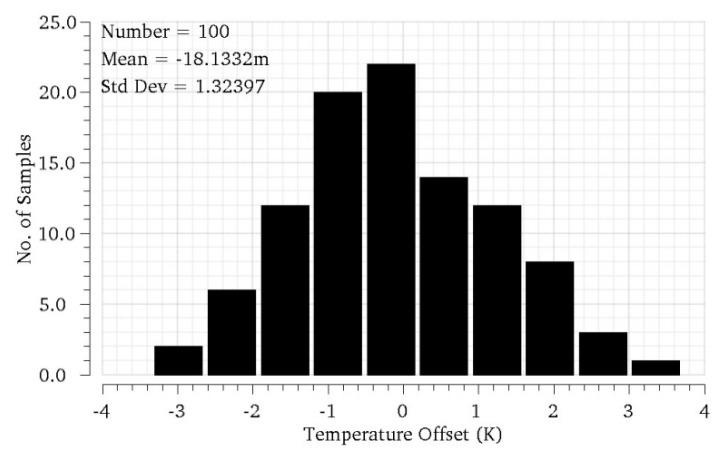
Histogram of the input-referred offsets of the core sensor circuit at balance, Δ*T_Q_* = 0 K, produced by manufacturing variability.

As stated in Section 3.4, bleeding currents should also compensate the effects of random manufacturing variability and other offset sources. In order to estimate the amount of offset produced by manufacturing variations, Monte-Carlo simulations of the core sensor in Figure 3 have been performed at *T_Q_*_1_ = *T_Q_*_2_ = 300 K, using MOS variability models provided by the manufacturer (process and mismatch). Note these mismatch models ignore the actual significant distance between transconductors Q1 and Q2 and thus may produce somewhat optimistic results. Figure 11 shows the histogram of the input-referred offsets in the characteristic curve obtained after 100 simulations of the circuit. The obtained histogram can be approximated to a Gaussian probability distribution with standard deviation σ = 1.33 K. In order to compensate manufacturing variability with a 99.99% yield, bleeding currents should cover offsets within ±3.9σ, which is about ±5.2 K. Note, this variability offset correction is in addition to the +10 K maximum temperature variation expected from the operation of the PA. Overall, bleeding currents should be designed to produce offsets from −5.2 to +15.2 K, with a 0.0 2K resolution. 

The need to compensate both positive and negative offsets, in principle, would require two different DAC circuits implementing two bleeding current sources. Positive offsets up to +15.2 K would be covered by a DAC, bleeding current to transistor Q2, as depicted in Figure 6, while negative offsets of up to −5.2 K would be covered by a second and smaller DAC, bleeding current to transistor Q1. In order to simplify the circuit and avoid the addition of this second DAC, a constant current source producing a constant offset of −5.2 K is added to transistor Q1. Therefore, a single DAC circuit, bleeding current to transistor Q2, will be sufficient to cover both the variability offsets and temperature variations. The DAC circuit must produce offsets comprised between 0 and 20.4 K. The full-scale current *I_FS_* must produce at least this 20.4 K offset, then:

(19) IFS≥ΔTmax·SBJT≈ΔTmax·IQ[μA]·40nAK=408·IBIAS[μA] nA

From Equations (18) and (19), the required number of bits of the DAC converter is thus,

(20) $$2^{N}=\frac{I_{FS}}{I_{LSB}}\geq \frac{408\cdot I_{BIAS}\left[\mu A\right]}{0.4\cdot I_{BIAS}\left[\mu A\right]}=1020\Rightarrow N\geq \log_{2}\left(1020\right)$$

Thus, a *N* = 10 bit current-steering DAC can cover the targeted range, regardless of the *I_BIAS_* current selected. Still, currents produced by the DAC converter will depend on *I_BIAS_*. To account for that dependence and be able to tune *I_BIAS_* in the core sensor (in order to control the BJT thermal transconductance), the bleeding currents of the DAC will be obtained by mirroring from the *I_BIAS_* reference, with a ratio (see Equation (19)):

(21) $$\frac{I_{FS}}{I_{BIAS}}=0.408$$

## 5. Implementation of the Temperature Sensor to Monitor Aging Degradation of a RF Power Amplifier

### 5.1. Power Amplifier

**Figure 12:**
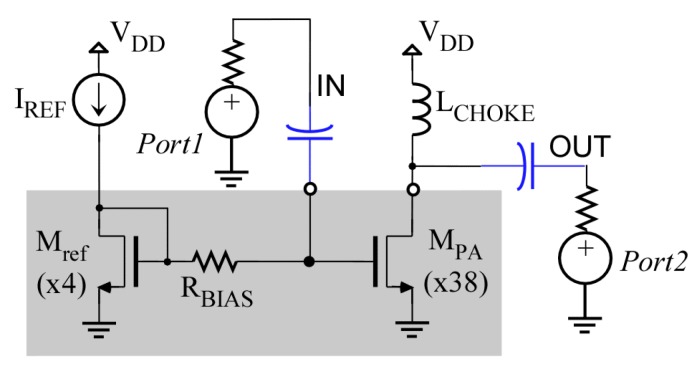
Scheme of the Power amplifier circuit used as a CUT. Greyed region indicates components integrated in the IC.

The above-described Temperature Sensor, together with an RF power amplifier (PA) as a CUT, has been designed and implemented in a 0.35 µm CMOS technology. The topology chosen is a single-transistor, single-stage, common-source class-A power amplifier, loaded with an off-chip choke inductor. The schematic of this PA is shown in Figure 12. The *M_PA_* MOS transistor is formed by a 38-unit transistor of dimensions 25/0.35 µm (each transistor gate has five fingers of *W* = 5 µm) connected in parallel. The nominal figures of merit of the PA include power gain, frequency of operation and maximum output power, which are presented in Table 3. External networks (not shown in the figure) provide input and output impedance matching 50 Ω in the frequency range of operation.

### 5.2. Temperature Sensor with Bleeding Sources

The temperature sensor consisting of the core circuit in Figure 3, plus a DAC acting as bleeding current source, has been designed in 0.35 µm CMOS. Lateral PNP transistors provided by the silicon foundry are used as transconductors Q1 and Q2. The output voltage of the sensor is buffered to the output pin through an OpAmp-based voltage follower. As described in Section 4, a forced imbalance in the sensor core will allow a single 10-bit current-steering DAC to produce both the positive and negative-input-referred offsets. The temperature variations will be measured from the value of the digital input of the DAC necessary to move the output voltage back to the quiescent value *V_OUT,Q_*, with an error not larger than the targeted resolution. Such a measurement procedure will be implemented with the help of an additional circuitry that, monitoring the output voltage, controls the DAC digital input in a feedback control loop. An essential characteristic for the DAC converter is thus monotonicity of its static transfer function, i.e., an always-positive slope. DAC converters implemented with switched binary-weighted elements are prone to non-monotonicity, especially when switching the most significant bits, as a consequence of manufacturing mismatches. A basic solution to guarantee monotonicity is to implement the DAC converter with unary elements— in this case, unary current sources all equal to the *I_LSB_*—which are enabled or disabled monotonically with the value of the input code [39]. A binary to thermometer decoder is thus required, but in spite of this added complexity, such unary-weighted solution will ease the implementation of the DAC in a regular layout, while also minimizing the effects of mismatch [40]. 

**Figure 13:**
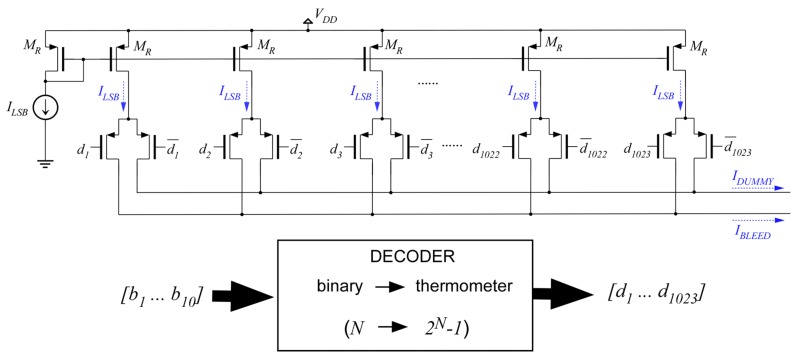
Scheme of the 10-bit current-steering DAC designed for the implementation of the bleeding current *I_BLEED_(n),* based on unary-weighted current sources.

Figure 13 shows the scheme of the DAC circuit that was, ultimately, implemented. The 1023 current sources are implemented with PMOS current mirrors with a 1:1 copy ratio with respect to a common reference current, and then are enabled to the output through switches controlled by the thermometer code. To avoid turning the current sources on/off, complementary switches to a dummy output are added [39]. Systematic INL (Integral Non-Linearity) of the DAC circuit produced by non-linear current dependence with its output voltage—finite output resistance—has been checked to be below ½LSB, while the regular layout and transistor dimensioning have allowed for minimization of the non-linearity produced by component mismatch. 

### 5.3. Layout

**Figure 14:**
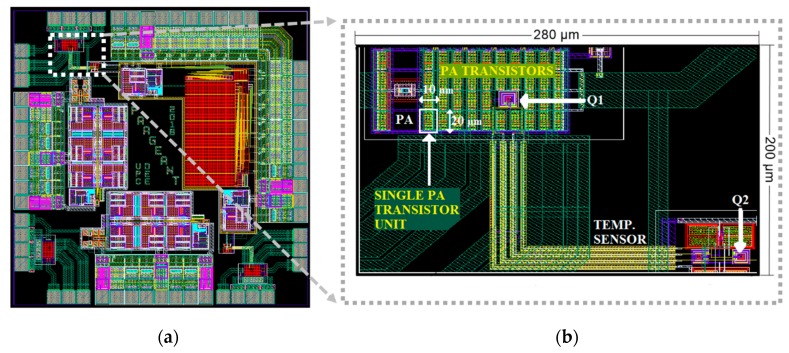
(**a**) Layout of the IC designed, containing several instances of the DUT + Temperature Sensor. (**b**) Detail of one of the power amplifiers and temperature sensor, highlighting the location of the temperature transducers Q1 and Q2.

Figure 14 shows the layout of the IC designed, together with a detail of the PA and the core of the temperature sensor. The overall integrated circuit has an area of 2000 μm × 2000 μm and includes several instances of the CUT and sensors, as well as output buffers and auxiliary circuitry. The white square in the detailed view of the layout highlights one of the 38-unit cell transistors that constitute PA transistor M_PA_ in Figure 12. To guarantee controlled current densities, these devices are placed in a matrix-like way (four rows and 10 columns). There are two voids within this matrix: row 3, columns 6 and 7. In this area, we have placed the bipolar transducer Q1. This bipolar transistor is a parasitic lateral PNP transistor, whose layout and model are provided by the design kit. Transistor Q2 is in the lower-right corner of the rectangle. This device placement implies that when the PA transistor dissipates power, transistor Q1 will always be hotter than transistor Q2. 

### 5.4. Example of Use of the Differential Thermal Sensor to Monitor Aging

**Figure 15:**
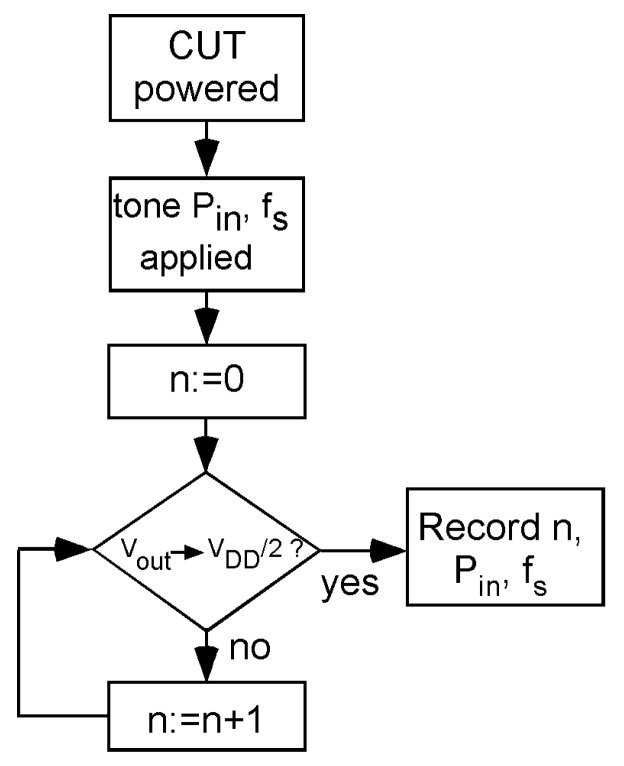
Algorithm to monitor aging in the PA using the thermal differential sensor with the homodyne technique.

In order to illustrate the use of the differential thermal sensor to monitor aging of the PA circuit, we consider the homodyne technique [10]. The algorithm to obtain a sensor output when a single tone is input to the PA is shown in Figure 15. 

First, the PA (or CUT in general) is powered, and next, a single tone at frequency *f_s_* and power *P_in_* is applied as its input. Frequency *f_s_* can be the central frequency of the PA but, in general, can be any frequency inside the bandwidth of the PA. Regarding power *P_in_*, it should maintain the amplifier in its linear region (below compression)—the larger the better. Next, the input digital word *n* of the DAC is zeroed, and a loop is applied: if the sensor’s output is *V_DD_*/2 (strictly speaking, inside the margin indicated by Equation (15)), then *n* is the value that indicates the differential temperature between the CUT and the sensor. If it is not, then *n* is increased. The loop is so simple thanks to the monotonicity of the sensor system, since the sensor has been unbalanced in such a way that in the worst case (process variation plus maximum temperature difference expected) the sensor’s output will initially be 0 V. 

Once *n* is obtained, it is recorded (together with the frequency *f_s_* and the applied RF input power *P_in_*). Note, this *n* is independent of ambient temperature that affects the IC as a whole. After a given operation time of the CUT (for aging degradation, this can be a long period), the algorithm is applied again with the same *P_in_* and *f_s_* conditions. If after this period, the CUT has suffered some aging degradation, then the DC power dissipated in the PA changes and so do the temperature sensed and the *n* output value. As *n’* is the new digital code that sets the output to *V_DD_/2*, then the temperature variation detected by the sensor can be obtained from Equations (7) and (16) as follows:

(22) $$\Delta P_{DC}=\frac{\Delta T_{Q}}{R_{THD}}=\frac{I_{BLEED}\left[n^{\prime }\right]-I_{BLEED}\left[n\right]}{R_{THD}\cdot S_{BJT}}$$

In this equation, the quantitative power variation corresponding to the difference between *n* and *n’* depends on the sensitivity of the bipolar transistor and on the thermal coupling resistance that, although they were estimated with simulation analysis, are not precisely known for each IC sample. Depending on the characteristic of the CUT to be monitored, this may require an additional previous characterization step. For example, to obtain—and monitor—the central frequency of this RF amplifier, obtaining the quantitative power is not necessary. Simply, the algorithm in Figure 15 would be applied for several input frequencies *f_s_*, with the purpose to identify the frequency at which the maximum *n* is produced [10]. On the contrary, if the RF output power is to be monitored, the quantitative relationship between the actually dissipated DC power in the CUT and the sensor reading is necessary. To obtain this relationship, a one-time previous characterization of the PA-sensor system would be required, simply measuring the sensor output for several DC power dissipations in the PA.

## 6. Conclusions 

Built-in temperature sensors are an attractive solution to monitor the performance of microelectronic analog circuits, particularly those operating at radio frequencies, because they are electrically non-invasive and because of the intrinsic down-conversion of the high-frequency information to a more relaxed low-frequency domain. The principles and basic techniques for thermal monitoring have been reviewed in this paper, namely the DC, homodyne—one tone—and heterodyne—2 tones—approaches. In particular, the last two techniques can be used to monitor the high-frequency AC performance of the CUT, with a low-frequency measurement.

A critical aspect for the successful application of these thermal monitoring techniques is, as can be expected, the design of the thermal sensor circuit; while using single-ended sensors based either in MOSFET or BJT transistors is possible, differential sensors are preferred because of their insensitivity to common-mode (e.g., ambient) thermal variations, among others. In order to sense small temperature differences produced by variations of the power dissipated in neighboring analog circuits, the sensor circuit should provide a high sensitivity, which results in a reduced dynamic range. However, a large range of detectable temperatures is also a critical requisite for the sensor, because it will suffer high offsets produced by either DC power dissipation of even manufacturing variability.

A systematic methodology to design a temperature sensor with both high sensitivity and high dynamic range is presented in this paper. A digitally controlled current source—a current-steering DAC—can provide dynamic range extension and, ultimately, provide the temperature reading in the digital domain, after inserting the sensor in a control loop. Electro-thermal simulations of the physical layout of the temperature transducer and the CUT allow the targeted sensor resolution and dynamic range to be set. This range is then extended to account for the effects of manufacturing variability. Based on this information, the complete sensor, including the digitally controlled offset correction, is dimensioned. The above methodology is applied to the design of a temperature sensor for monitoring the aging degradation produced in an RF power amplifier. Finally, the physical design of a circuit integrated in a CMOS 0.35 µm technology, containing the temperature sensor and circuit under test, is described.

## Figures and Tables

**Table 1:** Comparison of different differential temperature sensor implementations.

Reference	Technology	Transducer Device	CUT	Figure of Merit Monitored	CUT Driving Technique
[23]	1.5 µm, BiCMOS	Bipolar	Digital	Current consumption	Transient measurement
[34]	0.18 µm, CMOS	Parasitic vertical	LNA, 1.7 MHz	1dB Compression point	Homodyne
[30,31]	65 nm CMOS, triple well	Parasitic vertical	Power amplifier, 2-2.5 GHz	Output power, efficiency	Homodyne
[11]	0.25 µm, BiCMOS	Bipolar	LNA, 2.4 GHz	Structural test	Homodyne, DC measurements
[10]	65nm CMOS, triple well	Parasitic vertical	Power amplifier, 60 GHz	Central freq., 3dB bandwidth	Homodyne
[32]	65nm CMOS, triple well	Parasitic vertical	Power amplifier, 60 GHz	Central freq., 3dB bandwidth	Heterodyne
[35]	0.35 µm, CMOS	Parasitic lateral	MOS transistor		Homodyne
[36]	0.25 µm, CMOS. Triple Well	Parasitic vertical	LNA, 1GHz	Central freq., 1dB compression point	Heterodyne

**Table 2:** Temperature Sensor Performance.

	*I_BIAS_* = 1 µA	*I_BIAS_* = 6 µA
Sensitivity (V/K)	8.9	6.5
Bandwidth (kHz)	11.5	46.12
Slew Rate (kV/s)	11.3	45.8

**Table 3:** Power amplifier performance.

	Value
Power Supply Voltage (V)	3.3
Operating Frequency (GHz)	2.45
Power Gain (dB)	6.9
P_1dB_ (dBm)	13.5
